# Editorial: Advances in Musculoskeletal Ultrasound

**DOI:** 10.3389/fresc.2022.839819

**Published:** 2022-03-04

**Authors:** Wen-Shiang Chen, Chueh-Hung Wu, Mathieu Boudier-Revéret

**Affiliations:** ^1^Department of Physical Medicine and Rehabilitation, National Taiwan University Hospital, College of Medicine, National Taiwan University, Taipei, Taiwan; ^2^Department of Physical Medicine and Rehabilitation, Centre Hospitalier de l'Université de Montréal, Montréal, QC, Canada

**Keywords:** ultrasound, muskuloskeletal, fascia, shoulder, dysphagia, sonoelastography, tendinopathy

In this Research Topic entitled “Advances in Musculoskeletal Ultrasound” published in the rehabilitation for musculoskeletal conditions section of Frontiers in Rehabilitation Sciences, we present four articles from authors around the globe that illustrate the importance of ultrasound in diverse rehabilitation practices.

As more and more residency programs involved in rehabilitation are incorporating ultrasound in their curriculum (1, 2) and as many practicing rehabilitation professionals are getting trained in both basic and advanced ultrasonography (3, 4), the applications of this technology within our field increase.

The four articles in this Research Topic examine the body from head to toe; looking at the muscles of mastication and their association fasciae in detail in healthy volunteers; reviewing the use of ultrasound in the assessment of dysphagia; appraising the most recent evidence on the use of sonoelastography for shoulder soft tissue disorders; and evaluating the correlation between ultrasound biomarkers and clinical signs and symptoms in patients with chronic midportion Achilles' tendinopathy.

The original article *Ultrasound Imaging of Head/Neck Muscles and their Fasciae: an Observational Study* obtained normative data of ultrasound imaging of the fasciae of the masseter, temporal and sternocleidomastoid muscles (Pirri et al.). Sixteen healthy subjects were scanned to evaluate the thickness and variation in size with contraction of masticatory muscles and their associated fasciae. This lays the ground for future work exploring potentially significant asymmetry.

In the review article *Emerging Role of Ultrasound in Dysphagia Assessment and Intervention: A Narrative Review*, the role of ultrasound to evaluate tongue and hyolaryngeal movement, submental muscle contraction, upper esophageal sphincter, and airway structures, is described (Hsaio et al.). Given its inherent advantages of non-invasiveness and portability, ultrasound can complement video fluoroscopy and can also help interventions such as guided muscle injections for oropharyngeal spasticity.

In the mini review on *Sonoelastography of the Shoulder: A Narrative Review*, the authors analyze 19 articles published since 2018 using sonoelastography to assess shoulder tendons or ligaments (Babaei-Ghazani et al.). It allows *in vivo* assessment of qualitative and quantitative biomechanical properties of various tissues and has multiple potential applications in the musculoskeletal system, although it is not yet used routinely in practice.

Finally, in the original article *To What Extent Do Musculoskeletal Ultrasound Biomarkers Relate to Pain, Flexibility, Strength, and Function in Individuals with Chronic Symptomatic Achilles Tendinopathy?* 41 subjects with unilateral midportion chronic Achilles' tendinopathy had a battery of physical tests, and pain and function questionnaires were administered (Lalumiere et al.). Ultrasound examination of bilateral Achilles' tendons was performed, and the images were further analyzed by a specifically designed MATLAB program (5) that evaluated regions of interest within the tendons for geometric, composition, and texture biomarkers. Significant differences between the ultrasound biomarkers were found between healthy and pathological tendons. However, the correlation coefficients between the ultrasound biomarkers and the clinical signs and symptoms were negligible.

We hope readers will enjoy these four articles and that they will be stimulated to push the use of ultrasound in rehabilitation even further for better patient care.

## Author Contributions

All authors listed have made a substantial, direct, and intellectual contribution to the work and approved it for publication.

## Conflict of Interest

The authors declare that the research was conducted in the absence of any commercial or financial relationships that could be construed as a potential conflict of interest.

## Publisher's Note

All claims expressed in this article are solely those of the authors and do not necessarily represent those of their affiliated organizations, or those of the publisher, the editors and the reviewers. Any product that may be evaluated in this article, or claim that may be made by its manufacturer, is not guaranteed or endorsed by the publisher.
